# A Novel Solid Media-Free In-Planta Soybean (*Glycine max*. (L) Merr.) Transformation Approach

**DOI:** 10.3390/life14111412

**Published:** 2024-11-01

**Authors:** Muhammad Waqar Khan, Aaqib Shaheen, Xuebin Zhang, Yaser Hassan Dewir, Nóra Mendler-Drienyovszki

**Affiliations:** 1 State Key Laboratory of Crop Stress Adaptation and Improvement, Henan Joint International Laboratory for Crop Multi-Omics Research, School of Life Sciences, Henan University, Jinming Road, Kaifeng 475004, China; waqar@henu.edu.cn; 2 State Key Laboratory of Crop Stress Adaptation and Improvement, College of Agriculture, Henan University, Kaifeng 475004, China; aaqibtoshaheen@gmail.com; 3 Plant Production Department, College of Food and Agriculture Sciences, King Saud University, Riyadh 11451, Saudi Arabia; ydewir@ksu.edu.sa; 4 Research Institute of Nyíregyháza, Institutes for Agricultural Research and Educational Farm (IAREF), University of Debrecen, 4400 Nyíregyháza, Hungary; mendlerne@agr.unideb.hu

**Keywords:** *Agrobacterium*-mediated transformation, Fabaceae, in-planta regeneration

## Abstract

Soybean’s lengthy protocols for transgenic plant production are a bottleneck in the transgenic breeding of this crop. Explants cultured on a medium for an extended duration exhibit unanticipated modifications. Stress-induced somaclonal variations and in vitro contaminations also cause substantial losses of transgenic plants. This effect could potentially be mitigated by direct shoot regeneration without solid media or in-planta transformation. The current study focused primarily on developing a rapid and effective media-free in-planta transformation technique for three soybean genotypes (Wm82) and our newly developed two hybrids, designated as ZX-16 and ZX-3. The whole procedure for a transgenic plant takes the same time as a stable grown seedling. Multiple axillary shoots were regenerated on stable-grown soybean seedlings without the ectopic expression of developmental regulatory genes. An approximate amount of 200 µL medium with a growth regulator was employed for shoot organogenesis and growth. The maximal shoot regeneration percentages in the Wm82 and ZX-3 genotypes were 87.1% and 84.5%, respectively. The stable transformation ranged from 3% to 8.0%, with an average of 5.5%. This approach seems to be the opposite of the hairy root transformation method, which allowed transgenic shoots to be regenerated on normal roots. Further improvement regarding an increase in the transformation efficiency and of this technique for a broad range of soybean genotypes and other dicot species would be extremely beneficial in achieving increased stable transformation.

## 1. Introduction

The use of effective genetic transformation techniques in plants plays a crucial role in the development of agricultural biotechnology, facilitating the incorporation of novel characteristics such as disease resistance, increased drought tolerance, or higher nutritional values [1]. Biolistic DNA methods have been developed to cross the living cell barriers without involving the host–pathogen phenomenon. To obtain high transformation, a modified protocol of particle bombardment integrated with *agrobacterium* inoculation was developed [2]. However, this protocol also required a prolonged period of time for transformed cell proliferation and differentiation in whole stable transgenic plants in a culture medium. The use of solid media for this purpose prolonged the process of transgene production as compared to simple *Agrobacterium tumefaciens*-mediated transformation. The acquired transformation in soybeans had not been achieved while attempting these protocols [3]. However, some advancement in this field was seen after the discovery of nanotechnologies. The direct delivery and expression of foreign DNA into plants have been accomplished through nanoparticles. The high transient expression was achieved with a low, stable transformation [4,5]. Moreover, the discovery of RNA viruses for the delivery of sgRNAs with an extra sequence promoted cell-to-cell movement [6]. However, one of the drawbacks of this TRV vector was that it could not be used for a large gene delivery in the CRISPR system [7]. The hairy root transformation protocols were developed to acquire transiently expressed transgenes without a growth medium. The transgenic roots have been developed directly on stable-grown seedlings transformed with *Agrobacterium rhizogenes* [8,9,10].

In order to obtain genetically altered plants, in vitro plant propagation is a prerequisite. In the initial step, suitable growing conditions define the cells fate through reprogramming, differentiation, and morphogenesis. All these events lead to the transformation of cells or tissues into stable transgenic plants. In these conditions, the solid growth medium plays a vital role. It provides an aseptic environment for contamination-free transgenic plant acquisitions. Solid media are paramount for in vitro plant propagation because they provide the essential growth factors, important hormones, and energy sources [11]. The use of culture media helps in cell proliferation for somatic embryo reproduction and accelerates the growth of regenerated shoots. However, the addition of certain media components (growth regulators, sucrose, or MS) in a plant culture medium has some limitations in addition to their benefits. Furthermore, in vitro plant propagation in a culture medium is a rigorous procedure that requires great care for material handling. The incorrect and frequent use of solid growth media together with various types of hormones for an extended period of time causes oxidative stress, leading to chromosomal aberrations and other unexpected changes in transgenic plants. Transferring plants from one medium to another also inhibits the growth of ex-plants and causes some stress effects until they adopt a fresh media environment. These practices also prolonged the life span of first-generation transgenes from 8 weeks to 36 weeks [12]. Even genetically unmodified plants cultivated on solid culture media showed extended somaclonal variations [13,14,15,16,17]. To reduce the somaclonal variation, a simple and direct shoot organogenesis protocol was developed. Soybean transgenic shoots were obtained directly from the cotyledonary node explant without callus generation [18]. In comparison to previous culture media procedures, the direct organogenesis following an agrobacterium-mediated transformation also decreased the time for the acquisition of transgenic plants. Most recently, in-planta transformation gained momentum in transgenic plant production while mitigating the risk of somaclonal variations. In *Arabidopsis*, this protocol requires only bacterial culture and inoculation media for the direct transfer of T-DNA to floral parts through the floral dip approach [19]. No solid media culture was required for tissue or shoot regeneration. Based on this procedure, many attempts have been made to transform higher plants; however, only a model plant such as alfalfa (*Medicago truncatula* Gaertn.)*,* oilseed radish (*Raphanus sativus* L.), and some other plants have been transformed successfully. No evidence of other economically important plant transformations through the floral dip method has been reported [20].

Soybean (*Glycine max*. (L.) Merr.) is one of the most important nutritionally and economically as well as protein-rich crops originating from Asia and belongs to the Fabaceae family [21]. It has high nutritional values, so it can be used as food, feed, and biofuel. Conventional breeding methods are of great importance in soybean; however, the introduction and application of new breeding tools and methods can overcome the barriers for simple and fast soybean breeding [22,23]. Recently, a single plasmid was used to deliver gene-editing and developmental regulators to plant tissues for transient and stable transformations [24]. They transformed the binary vector into axillary buds and leaf disks of stable-grown tobacco to obtain transgenic shoots. However, the unregulated expression of developmental regulators had a negative impact on phenotypes. The main aim of our research was to develop a novel in-planta shoot regeneration and transformation method by using three soybean genotypes as the experimental material. This study describes a rapid, robust, cost-effective, and simple protocol for shoot induction and transformation. The protocol does not involve solid media for shoot or root induction, an aseptic environment for bacterial inoculation, or most of the material handling. Moreover, there was no need to express endogenous developmental regulators inside transgenic cells. The *agrobacterium* was transformed into the cotyledonary node portion of the stable-grown soybean seedlings, and a combination of growth regulators was applied exogenously to promote shoot induction. Initially, a composite transgene (transgenic shoots with a non-transgenic base) could be obtained in the T_0_ generation. However, if needed, the positive shoot can be simply rooted in the soil pots to have whole transgenic seedlings in the T_0_ generation. However, this protocol is in the stage of its infancy; further investigation is needed to boost the transformation efficiency and stable soybean production of recalcitrant plants.

## 2. Materials and Methods

### 2.1. Seed Preparation and Seedling Growth

Soybean (*Glycine max*. (L.) Merr.) seeds from three genotypes (William82 (Wm82) as Wm82 are susceptible soybean genotype and easy to transform, and our newly developed two hybrids, designated as ZX-16 and ZX-3, provided by Henan University, Zhengzhou, China) were surface sterilized using 70% ethanol for 5 min followed by 4% sodium hypochlorite (NaOCl) for 14 min. The seeds were rinsed with distilled water before being planted in pots containing a sterile mixture of vermiculite and peat (1:1; *v*/*v*). Two seeds were planted in each pot and incubated under a controlled environment (25 °C, 60% white florescence light, and 13/11 (day/night) hours of photoperiod with 60% relative humidity (RH)). Following germination, the seedlings were allowed to grow when the cotyledonary leaves fully expanded. Unhealthy and contaminated seedlings were carefully discarded. After six days of germination, the excision and inoculation of the explants were carried out.

### 2.2. Excision of Hypocotyls and Agrobacterium Infiltration

**Figure 1:**
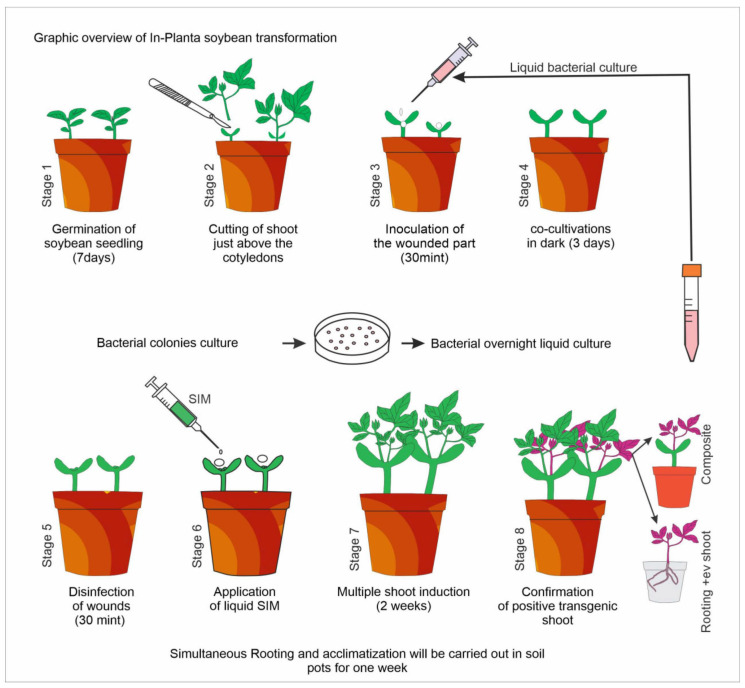
Graphic overview of the in-planta soybean transformation protocol. This protocol is divided into different stages. The whole process will be accomplished in eight short steps. The total duration from seed germination to 5 cm of putative transgene will take almost 4 weeks.

The stems from the explants above the cotyledons were excised using a sterile blade. A day before inoculation, *A. tumefaciens* harboring the GmUbi-3XFlag-GUS: GFP vector was cultured (28 °C, 12 h) in yeast extract peptone (YEP) media with selective antibiotics. The overnight bacterial culture was centrifuged at 4000 rpm for 10 min. The cell pellet was re-suspended to an OD_600_ (optical density at 600 nm) of 0.4 in infiltration media (1/2 strength Murashige and Skoog (MS) medium [25] with vitamins, 2% (*w*/*v*) sucrose, 200 µM acetosyringone, and 250 mg/L L-cysteine, pH 5.4). The bacteria culture medium was incubated at room temperature for 30 min. Prior to the inoculation of the explant, all tools were surface sterilized with 75% ethanol. The axillary buds on either side of the stem were removed. Close to the cotyledon base, several vertical cuts were made. Detailed procedure is given in Figure 1. A small quantity (200 µL) of infiltration media was poured over each explant’s wounded portion. The light over the explants was switched off for 3 days. The inoculated areas were routinely kept moist with co-cultivation medium (1/2 strength MS medium with vitamins, 2% (*w*/*v*) sucrose, 200 µM acetosyringone, 250 mg/L L-cysteine, 0.3 mg/L gibberellic acid (GA_3_), 2 mg/L spermidine, and 1.3 mg/L indole butyric acid (IBA), pH 5.4) during the three days of co-cultivation under dark conditions.

### 2.3. Vector Assembly and Cloning

For the construction of the GUS expression vector, computer simulation was performed in Snapgene software version 6.0.2 by using GmUbi-3XFlag-GFP as the destination vector (Figure S1B). The GUS fragment was amplified from the pCambia-1300 vector by using a pair of overlapping PCR primers: the forward primer GACTCGACAGTCTAGAATGGGTTTACGTCCTGTAGAAACC and the reverse primer TCCTTATAGTCCATGGTACCTCATTGTTTGCCTCCCTGCTGC (other related primers list is given in Supplementary Table S1). Double digesting the vector with Asc I and Xba I restriction endonucleases linearized the vector. Digestion of the vector was carried out in a 50 µL reaction volume containing 1 µg plasmid, 1 µL each of enzyme, 5 µL quick-cut buffer, and ddH_2_O up to 50 µL volume. The reaction mix was incubated at 35 °C for 2 h. The products were resolved on 0.8% agarose to confirm the digestion of the vector. The digested vector band was purified from the gel using gel extraction kit. The digested vector was allowed to ligate with the GUS fragment containing a complementary overhang. Ligation of the GUS gene cassette and GmUbi-3XFlag vector was performed using a one-step DNA cloning kit (Novoprotein, Shanghai, China), following the user’s manual. The ligated vector was transformed into a competent bacterial cell by the heat-shock method. The selection of true colonies was made through colony PCR and then the true ligated GUS gene cassette was confirmed through Sanger sequencing. The positive colonies were cultured overnight in LB liquid media with selective antibiotics. The vector was extracted from the bacterial colonies by suing plasmid extraction kit. The vector was then transformed into Agrobacterium GV3101 by the freeze–thaw method. The positive *A. tumefaciens* was cultured in YEP media with selective antibiotics overnight. The overnight culture was mixed with 30% glycerol (1:1) and stored in −80 °C freezer for future use.

### 2.4. Washing and Shoot Induction of the Inoculated Part

The inoculated explants were washed with distilled water after three days of co-cultivation to remove the adherent agrobacterial culture and media residue. The residual *A. tumefaciens* were effectively removed by washing the wounded parts with washing media containing ½ MS supplemented with 250 mg/L Cefotaxime and 250 mg/L Carbenicillin for 30 min. The explants were then subjected to shoot induction under 13/11 h of light/dark conditions and 70% RH. We used the modified Fast-TrAAC protocol by Maher et al. [24]. For fast shoot regeneration, we used our modified shoot induction media [26]. The shoot induction medium (SIM) contained full-strength MS medium with vitamins, 0.2 mg/L MgCl_2_ 6H_2_O, 1.3 mg/L 6-benzyl aminopurine (6-BA), 2.5 mg/L spermidine, 0.4 mg/L (N6-(2-isopentenyl) adenine (2iP), 0.3 mg/L kinetin, 0.3 mg/L GA_3_, 150 mg/L Cefotaxime, 200 mg/L Carbenicillin, and 0.5 g/L 2-(N-Morpholino) ethane sulfonic acid monohydrate (MES). The pH of the medium was adjusted to 5.7. A small amount of SIM (200 µL) was poured over the inoculated portion for new shoot emergence. The air temperature and RH were kept constant to prevent fungal contamination. The seedlings were properly watered with dissolved water-soluble nitrogen fertilizers (2 g/L) for a constant supply of basic nutrients. The fertilizers were applied at a one-week interval until shoot harvesting. The newly emerged shoots from the inoculated portion were subjected to transgenic shoot identification when they reached 4 cm in length. The shoots that showed no GUS or GFP signals were considered non-transformed and were discarded. The explants were allowed to regenerate more axillary shoots.

### 2.5. Identification of T-DNA and Root Induction of Positive Transgenic Lines

For the transgenic identification, total genomic DNA was extracted from a putative transgenic line. While using the pMDC32 vector, the T-DNA cassette was identified through *hpt*-specific PCR using a pair of primers (forward ATTTGTGTACGCCCGACAGT and reverse CTCTCGGAGGGCGAAGAATC), followed by seedling selections in the T1 generation in hygromycin B-supplemented medium. An optimized PCR program was established to identify 840 bp of the *hpt*II gene in transformed soybean shoots. Moreover, an empty GmUbi-3XFlag-GFP: GUS vectors’ T-DNA was confirmed through PCR, GFP signals, and GUS histochemical analysis. For stable transgene production throughout the process, we used our optimized root induction strategy. The positive shoots were excised and rooted in pots containing a sterile mixture of vermiculite: peat (1:1; *v*/*v*), moistened with ½ MS liquid medium with vitamins, and 4 mg/L indole-3 butyric acid (IBA). The seedlings were covered with plastic humidity domes (Suzhou Huanmei Plastic, Suzhou, China). Over the course of one week, both the root induction and acclimatization were carried out. Most of the transplants were permitted to grow as composites with a single positive shoot.

### 2.6. GUS Histochemical Analysis and GFP Detection

After two weeks of regeneration, the shoots were subjected to GUS histochemical analysis. The cotyledon portion along with newly emerged shoots were analyzed for GUS expression. The GUS staining buffer was 50 mM NaH_2_PO_4_ (pH 7.2), 10 mM Na_2_EDTA, 0.1% (*v/v*) Triton-X100, 1 mM K_4_Fe(CN)_6_, 1 mM K_3_Fe(CN)_6_, and 2 mM X-gluc. The plant samples were incubated overnight (12 h) at 37 °C as described by Jefferson et al. [27]. The transformation efficiencies were calculated based on positive GUS events. For GFP identification, the T_0_ putative transgenic lines and then the T_1_ lines were subjected to GFP identification at different times. The GUS signals were detected through the confocal microscope LSM980 (ZEISS, Oberkochen, Germany). The GFP parameters were set to acquire clear images of the GFP florescence; the excitation wave length was 488 nm and the emission wave length was 509 nm. The detection of the images was carried out from 491–544 nm. Both the GFP and bright field images were acquired and processed in the ZEISS Efficient Navigation ZEN 3.1 software version.

### 2.7. Statistical Analysis

All experiments were set up in a completely randomized design with multiple biological replicates. The mean ± standard deviation was used to express the regeneration frequency, shoot induction rate, and transient transformation frequency. The percentage data were arcsine transformed before analysis, and mean values were compared using Tukey’s multiple range test in SAS software (version 9.4; SAS Institute, Inc., Cary, NC, USA).

## 3. Results

### 3.1. Outline of a Rapid In-Planta Soybean Transformation Methodology: An Innovative Approach

The soybean transgenic breeding process is time-consuming and lengthy. To shorten the time required for transgene acquisition and further simplify the process, we developed the in-planta soybean transformation protocol. The process began with seed germination in soil pots, followed by the inoculation of these explants in an open growth chamber. The entire protocol was divided into eight fundamental steps. During the first step, soybean seeds were sown in the soil pots, and the seedlings grew until their cotyledonary leaves expanded; details of the procedure are presented in Figure 1. Depending on the growth conditions and the type of soybean genotype used, the inoculation period varied. For bacterial inoculation, only a modest amount of 200 µL of bacterial liquid culture was required. The inoculated explants were retained in co-cultivation for two or three days. During this stage, co-cultivation liquid media were applied to keep the injured portion moist. A small quantity of this medium aided the *agrobacterium* in transferring its T-DNA into the cells. The inoculated parts were washed with distilled water and disinfected with MS media containing 250 mg/L each of cefotaxime and carbenicillin. The shifting of the explant inhibited new shoot emergence and growth. A small amount (200 µL or less) of SIM containing growth regulators were supplied to the infected portion. This procedure was repeated on a daily basis until the infected part formed somatic embryos. The SIM media was applied for a period of one week. For somatic embryogenesis and multiple shoot induction, one week is enough time. The shoots were allowed to grow further. The transgene was identified after the shoot reached a maximum length of 4 cm. The unsliced axillary buds, which produced a quick, extended shoot, was removed. Positive transgenic shoots will either grow as a composite on the main stem or will be separated out and rooted in soil posts.

For the speedy development of the transgenic plants, the single positive shoot allowed them to grow as composites. However, in the case of more than one transgenic shoot, an extra transgenic shoot was rooted separately. Rooting and acclimatization took place in soil pots at the same time. The soil was soaked with MS medium containing 4 mg/L IBA, and the transgenic stems were allowed to root for one week.

### 3.2. Experimental Evidence of the Novel In-Planta Soybean Transformation

Initially, the experiment was carried out using William82 soybean genotypes. The sterilized Wm82 seeds were planted in soil pots. After six days, the hypocotyls were removed, and the wounded areas were infected with *Agrobacterium* harboring the pMDC32 binary vector containing *hptII* as the reporter gene. The integrated *hptII* gene showed expression in the soybean transgenic lines under the CaMV-35S promoter (Figure S1C). This effect was confirmed through the selection of putative transgenic soybeans on hygromycin B-supplemented medium. The positive transgenic soybean lines induced roots on selective medium; however, non-transgenic shoots did not produce lateral roots even after two weeks of seedling culture. The root hairs were also inhibited by hygromycin B in non-transformed seedlings. For further verification, PCR amplification was performed. Seeing as the non-transformed control also produced a band following PCR amplification, the obtained fragments from transgenic lines were sequenced for confirmation. The Sanger sequencing confirmed the 840-bp *hptII* gene in the soybean genome in the T2 generation (Figure S1A,D).

**Figure 2:**
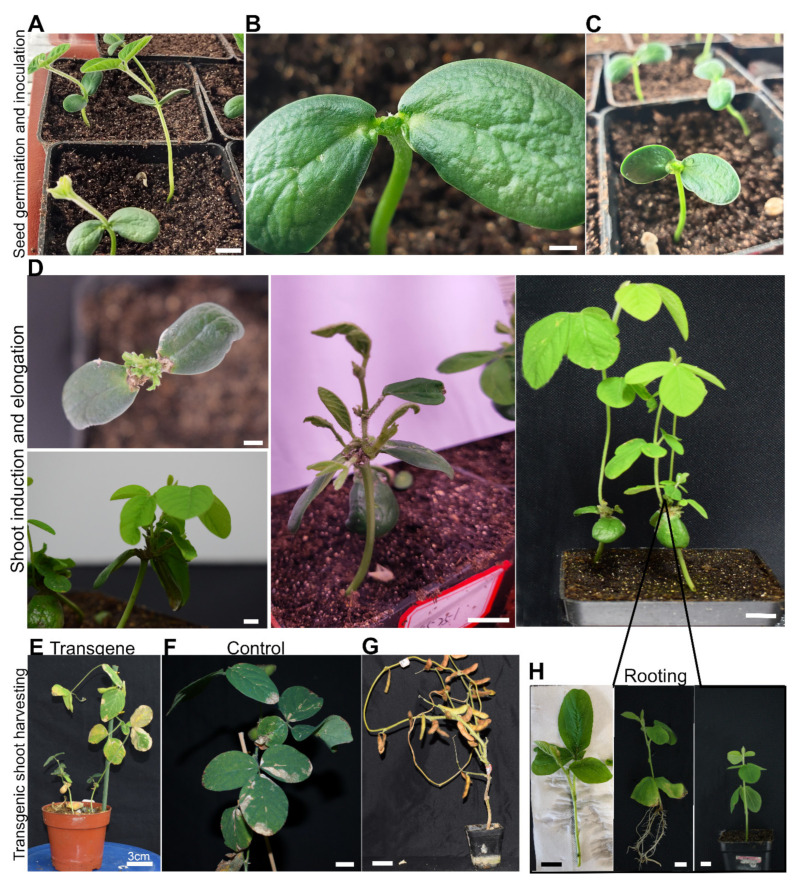
Composite soybean seedling regeneration from the cotyledonary node region of stably developed soybean seedlings. The entire procedure has been split into three major parts. Panels (**A**–**C**) represent the earliest stages of seed germination, inoculation, and co-cultivation. Panel (**D**) displays shoot induction, regeneration, and elongation, whereas Panels (**E**–**G**) depict transgene identification and T1 seed harvesting. Panel (**H**), on the other hand, shows the rooting of independent positive shoots in soil pots and the acclimatization of seedlings. The scale bars were set to be 3 cm.

After three days of co-cultivation, the adherent *Agrobacterium* was efficiently removed with a high concentration of antibiotics. The SIM was applied on a regular basis for the regeneration of the wounded parts. After one week of a daily dose of 200 µL of SIM, the explant produced a large number of somatic embryos. Each embryo developed into a shoot in a short time (Figure 2A–C). The fast shoot growth was observed due to the stable root system of the explant and the use of exogenous growth media supplemented with growth hormones. The application of water-soluble nitrogen fertilizers also played an important role in de novo shoot regeneration and growth. The new shoots reached a length of more than 6 cm within three weeks of culture on SIM (Figure 2D). No additional SIM was applied to the inoculated areas. Explants consisting of a single transgenic shoot were allowed to grow until harvesting. The explants that produced more than one positive transgenic shoot were removed and rooted in soil pots. These positive shoots were raised as an independent transgenic line (Figure 2H). The non-transgenic shoots were discarded, and the explant was allowed to regenerate. The time for T0 transgenic shoot harvesting was compared with the non-transformed seedling treated in the same way. Both transgenic and non-transformed stable shoots matured at the same time. This suggests that soybean transgenic plant acquisitions take an almost equal length of time for development. Furthermore, the transgenic shoots were productive compared to normal shoots. No sterile shoots were identified after the transformation of the stable seedling (Figure 2E–G).

### 3.3. Fast and Multiple Shoot Induction and Elongation on Stable Seedling

**Figure 3:**
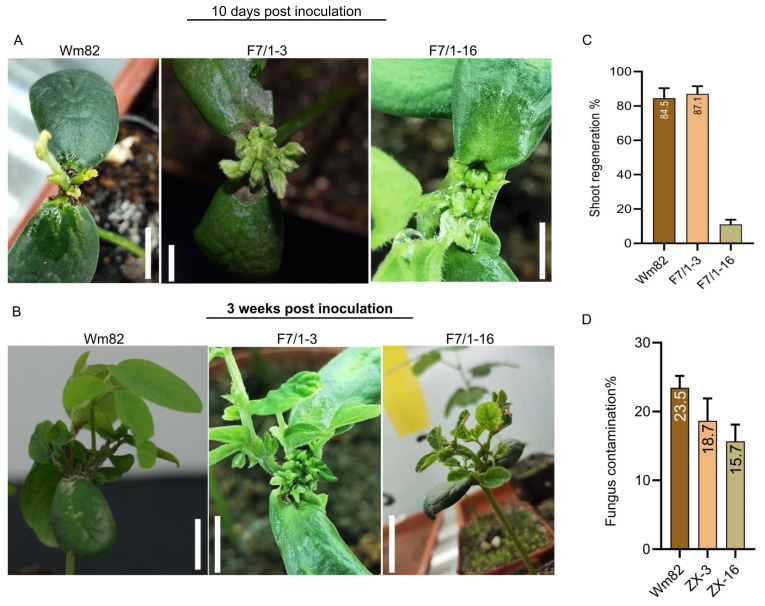
Three soybean genotypes’ shoot regeneration and elongation on stable growing explants. Panel (**A**) illustrates the regeneration of three soybean genotypes after 10 days, whereas Panel (**B**) indicates the regeneration after three weeks. For both panels, the scale bar was 3 cm. (**C**) Shoot regeneration percentage of three genotypes and (**D**) percentage of contamination of the three soybean genotypes following inoculation. Values followed by the same letter are not significantly different at *p* ≤ 0.05 level, according to Tukey’s multiple range test.

The basic stage in transgene formation is shoot induction. Multiple shoot inductions greatly influence the transformation efficiency of soybeans. To evaluate whether multiple shoot induction and transformation could be performed in other soybean genotypes, we included more genotypes in our investigations. For shoot regeneration and transformation, we introduced our newly developed soybean hybrids for shoot regeneration and *Agrobacterium*-mediated transformation. These genotypes were derived from a crossing between a Wm82-susceptible parent and a Chinese landrace LX. During the investigation, Wm82 was also included. Following the application of SIM supplemented with plant growth regulators (PGRs) as described above, all soybean genotypes developed numerous somatic embryos in the inoculated areas within one week. Somatic embryogenesis was higher in the two soybeans, Wm82 and ZX-3, than in ZX-16 after 10 days and three weeks of post-inoculation (Figure 3A,B). In Wm82, an average of 84.5% shoot regeneration was recorded, while in the ZX-3 soybean hybrid, it was 87.0%. ZX-16 showed the lowest response to shoot regeneration (11%) (Figure 3C). However, more shoots were investigated on ZX-16’s explant cultured on solid MS media. This implies that while employing this innovative in-planta shoot induction and regeneration, this response varied among the soybean genotypes. The shoot induction and regeneration responses were higher in the ZX-3 genotype when using in-planta shoot regeneration. However, this response of ZX-3 was inverse on solid MS media. Additionally, because of the open trail without any aseptic environment, there was a high risk of fungal contamination. Material handling during conventional shoot regeneration requires extreme caution. A minor mistake can cause a great loss of the experimental material and time. The recovery of the contaminants is also impossible with the solid media explant. During in-planta shoot regeneration, we observed some fungal contamination. This fungal contamination could simply be controlled by the use of autoclaved liquid medium and the partial sterilization of tools. However, the possibility of a fungal infection could not be eliminated completely. We documented fungal infiltration in explants following shoot regeneration after numerous tests. Based on the explant used and how the materials were handled, a limited spectrum of fungal contamination was identified. The degree of fungal contamination varied between the three genotypes. We calculated the number of infected explants divided by their total number. Wm82 explants had the highest contamination of 23.5%, followed by 18.7% and 15.7% in ZX-3 and ZX-16, respectively (Figure 3D). Some fungi, on the other hand, were easily eliminated from explants by washing them with tap water. After contamination, only a small percentage of the explants hindered their growth.

### 3.4. Efficient In-Planta Transgenic Soybean Plant Generation

**Figure 4:**
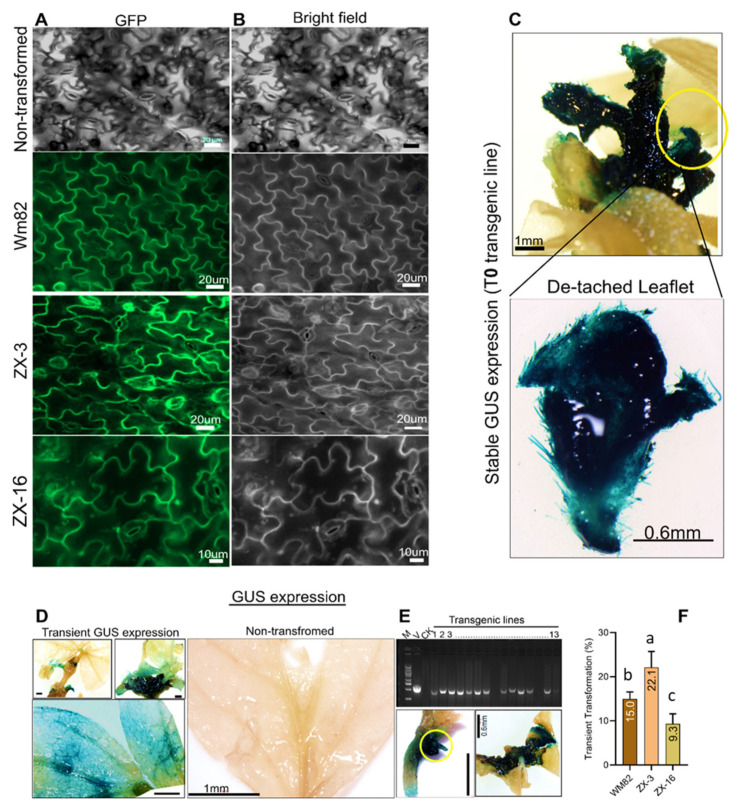
The transient and stable expression of GFP and GUS in three soybean genotypes transformed with *A. tumefaciens,* the harboring GmUbi-3xFlag-GUS vector. Panels (**A**,**B**) show the GFP and bright field images of the positive soybean lines from Wm82, ZX-3, and ZX-16. Panel (**C**) depicts the stable GUS expression in the T0 shoot; the lower part shows the detached leaflets from the stable shoot. The transient transformation of three soybean genotypes and a non-transformed control treated with GUS buffer are shown in Panel (**D**). During in-planta transformation: (**E**) the PCR amplified product of GUS gene in transgenic lines, where M indicates marker, V indicates vector control, CK is control, and numbers from 1 to 13 are transformed lines; (**F**) the graph indicates the average transient transformation of three soybean genotypes. Error bars show the deviation of average data from the mean value. Scale bars for different pictures are given inside the square box. Values followed by the same letter are not significantly different at *p* ≤ 0.05 level, according to Tukey’s multiple range test.

The GUS and GFP gene cassettes were transformed into three soybean lines through *A. tumefaciens* (GV3101) during separate events. The composite plants were grown until the putative transgenic shoots reached 4 cm in length. For the identification of the T-DNA section in the soybean genome, the total genomic DNA was isolated and PCR was performed. The GFP and GUS genes were identified using a pair of T-DNA-specific PCR primers, forward sequences 5′-GCCTCTTCGCTATTACGCCA and 5′-AATCATCGCAAGACCGGCAACAG. The primer pair identified a T-DNA section in newly emerged putative soybean shoots from inoculated sections (Figure 4E). The confocal microscopy images further verified the PCR-positive transgenic lines. The GFP fluorescence signals were detected in a large number of PCR-positive shoots. The bright field images were also acquired for a comparison of the false positive results (Figure 4A,B). All positive events were recorded, and the transient transformation was calculated based on positive GFP signals and PCR results. During a separate event, the transformed explants with the GUS expression vector were allowed for a GUS histochemical analysis (Figure S1E). The explant and putative transgenic shoot were subjected to overnight GUS staining. The explants from three soybean genotypes were incubated for 12 h in GUS buffer. After decolorization, the explants revealed transient GUS expression in distinct parts of the shoots. High GUS expression was detected in two soybean genotypes, ZX-3 and Wm82 (Figure 4D). This expression was lower in ZX-16 genotypes. Based on the GFP and GUS assay results, ZX-3 had the highest transient transformation rate of 21.1%, followed by Wm82 at 15.0% and ZX-16 at 9.3% (Figure 4F). Based on the ubiquitous expression of GUS in the shoot, the stable transformation in the T_0_ generation was determined (Figure 4C). The stable transformation efficiency during T_0_ was recorded as 5.5%. Although the stable transformation rate was low, this was still a very efficient soybean transformation protocol since we reduced the high cost of the solid media, the risk of ex-plant mortality, and the extended duration of transgene production on a solid medium.

## 4. Discussion

The availability of a rapid and rigorous technique for plant transgene development until the harvest stage remains a barrier. The embryogenesis of altered cells, followed by the morphogenesis into the whole plant, takes place in a stressful environment. Due to the numerous stress effects, many positive transgenic tissues would be wasted during the course. Progress has been made in the transformation systems of several plant species. In soybean, direct shoot regeneration was made possible from conditioned cotyledonary explants without callus formation [28]. The usage of diverse plant cultural mediums for callus induction and differentiation was minimized as a result of this shift. For the first time, transgenic shoots were successfully regenerated on the cotyledon explant using *Agrobacterium* as a gene delivery vehicle [8]. Since then, different modifications have been brought to the MS media composition for fast and multiple shoot regenerations. However, the tissue culture induces certain unwanted modifications in the quantitative traits of cultured transformants [29]. To minimize the likelihood of solid media-born mutations in targeted plants, a novel technique was recently devised. The CRISPR cassette and developmental regulators were cloned in a single vector and delivered to the axillary buds of stably growing plants using the novel Fast-TrACC method [24]. They were successful in creating a stable transgene; however, the uncontrolled and pleotropic expression of developmental regulators resulted in undesirable changes in phenotypes.

To overcome these deficiencies, we developed a novel soybean in-planta transformation protocol. We called this new protocol Fast-TraP (fast transgene production) as the T_0_ transgenic shoot could be obtained at the same time as normal-grown soybean. The composite-positive transgenic shoots in the T_0_ generation were identified using PCR, GUS analysis, and GFP florescence signals. We progressed by making our protocol more accessible to the scientific community at large. Instead of transferring genes for the regeneration of shoots through the expression of developmental regulators endogenously, as described, we used an exogenous combination of growth regulators for de novo shoot induction and regeneration on stable-grown soybean seedlings. The application of growth regulators facilitated the expression of endogenous genes for de novo shoot induction on the soybean cotyledonary node. This interplay of exogenous auxins and cytokinins was important in activating the expression of stem cell regulatory genes in the wounded part [30]. Meanwhile, different combinations of growth hormones and polyamines have been proven to induce *Agrobacterium* growth and transformation [31,32]. Thus, we used liquid growth media containing a combination of hormones and polyamines (spermidine) that not only promoted shoot regeneration, but also facilitated *Agrobacterium* transformation. In addition, the investigation of the fitness of a novel soybean in-planta protocol for diverse soybean genotypes revealed its applicability for all genotypes. A range of soybean genotypes, mostly susceptible, were the best sources for being easily transformed through this protocol because the soybean transformation efficiency is substantially genotype-dependent [33]. Another intriguing feature of this approach was the development of composite plants during T0 generation. The shoot-related attributes could be studied with a normal root system. It provides an opposite insight into hairy root transformation. *Agrobacterium rhizogenes* produced transgenic roots with wild shoots [34,35]. We foresee that this procedure will be more favorable, faster, and cost-effective in terms of producing composite or stable transgenes for functional or regulatory genomic studies in soybean. Moreover, other dicotyledonous plants could also be transformed by employing similar strategies. We conducted a separate experiment on chickpeas (*Cicer arietinum* L.) while using our novel in-planta transformation protocol. Similar procedures were adopted as described for soybean transformation. We identified some GUS signals in the leaves of the regenerated chickpea shoots. No further investigation was conducted on a broad scale. Nevertheless, the immune response of stable-grown soybeans or other plants may be stronger. Thus, the identification and use of a partial plant immune suppressor during inoculation may significantly improve the transformation efficiency. Moreover, the inclusion of various biochemicals or surfactants may also facilitate the *Agrobacterium* chemotaxis, transfer, and integration of T-DNA into the soybean. This phenomenon requires additional investigation.

## 5. Conclusions

The optimization of the in-planta shoot regeneration protocol without solid growth media reduced the risk of media-born somaclonal variation. These variations caused a substantial loss of desire for transgenic plants. Moreover, media-born contamination is also a basic problem in transgenic plant production. The stable seedling provided a natural combination of nutrients for fast regeneration and shoot growth. It also provided a novel insight into composite shoot studies on non-transformed root systems. The cost-effectiveness of this protocol cannot be denied. Only a small quantity of liquid inoculation and shoot induction media were required. Growing seedlings in soil also reduced the cost of disposable culture plates and other vessels. However, this protocol can be further improved by increasing the transformation percentage through the partial inhibition of the soybean immune response, the use of surfactants, and other transformation inducers. Further insight into the improvement of stable transformation through these protocols could revolutionize soybean genetic improvement.

## Data Availability

The original contributions presented in the study are included in the article; further inquiries can be directed to the corresponding author.

## Supplementary Materials

The following supporting information can be downloaded at: https://www.mdpi.com/article/10.3390/life14111412/s1, Figure S1: hptII transgene identification in transformed soybean lines. (A) The amplified PCR product of the hptII gene in soybean lines. (B) The GmUbi-3XFlag-GUS vector is used for transgene identification through a GUS histochemical assay. (C) The T-DNA section of the pMDC32 vector shows the hptII gene and its promoter. (D) The Sanger sequence results of the hptII gene identified in positive T2 transgenic soybean. (E) Reassembled circular vector; Table S1: A list of primers used during present investigations for cloning of vector and detection of T-DNA section.
