# Supplementary material for: A Novel Solid Media-Free In-Planta Soybean (Glycine max. (L) Merr.) Transformation Approach
Source: Life (Basel). 2024 Nov 1;14(11):1412. doi: 10.3390/life14111412 (PMC11595655; doi:10.3390/life14111412)
Supplement: Supplementary file 1 [file life-14-01412-s001.zip › life-3210349-supplementary.pdf]

A novel Solid Media Free In-Planta Soybean Transformation Approach

Muhammad Waqar Khan[1], Aaqib Shaheen[2], Xuebin Zhang[1]*, Yaser Hassan Dewir [3,] and Nóra Mendler-Drienyovszki[4]

<u>Supplementary files</u>

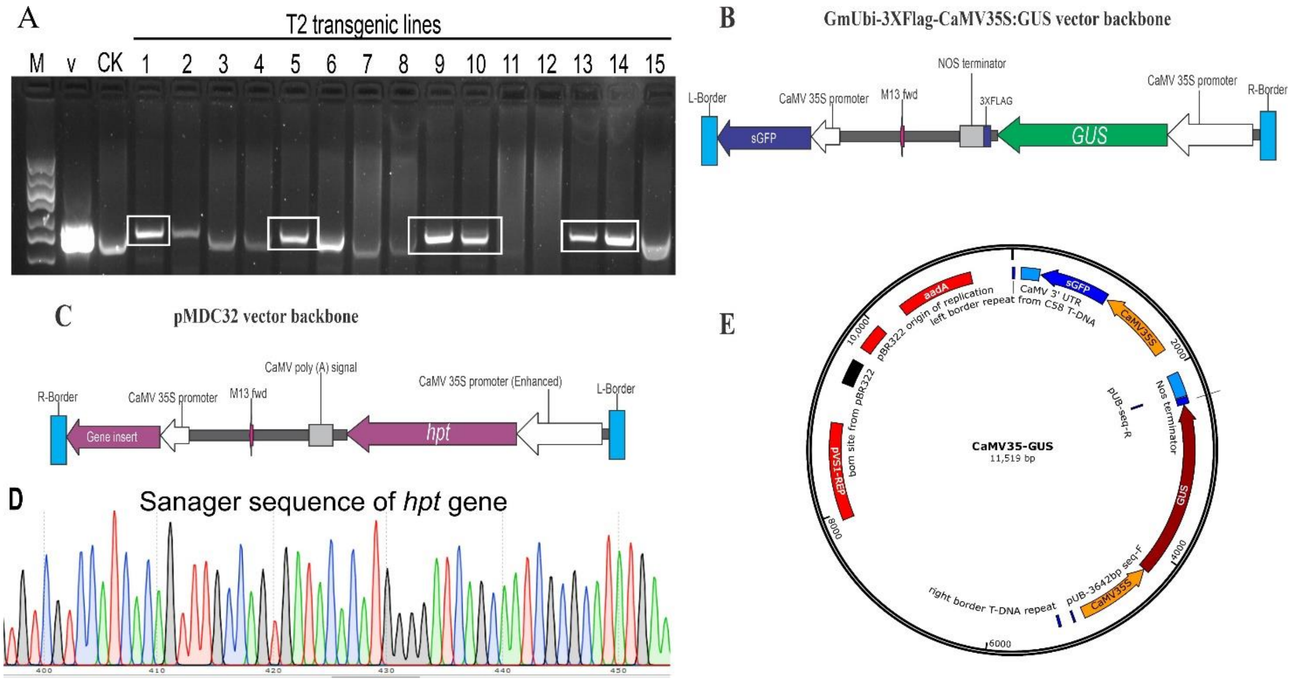

Figure S1. hptII transgene identification in transformed soybean lines A) The amplified PCR product of the hptII gene in soybean lines **B**) The GmUbi-3XFlag-GUS vector is used for transgene identification through a GUS histochemical assay. **C**) The T-DNA section of the pMDC32 vector shows the *hpt*II gene and its promoter. **D**) The Sanger sequence results of the *hpt*II gene identified in positive T2 transgenic soybean. **E**) Reassembled circular vector

Table S1: A list of primers used during present investigations for cloning of vector and detection of T-DNA section.

Primer's usage	forward (F) Oligo	Reverse (R) Oligo	Annealing temperature	PCR product
Camve-35S fragment cloning	**Camv-Fov** 5′-ACGTAAACCCATTCTA GAGAGATAGATTTGTAGAGAGA GA CTGGTGAT	**Camv-Rov** 5′-CCAGTGCCAAGCTGGGC ATGGTG GAGCACGACACTCTC	62°C	1.23kb
T-DNA detect primers	**GmUbi detect** 5′-ATTTTACAAATACA AATACATACTAAGGGTTTCT	**GmUbi detect** 5′-GCACCATCT TCTTCAAGGACG	56°C	300bp
GmUbi-T-DNA detection	5′-GCCTCTTCGCTATTACGCCA	5′-AATCATCGCAAGACCGG CAAC AG	55°C	320bp
CaMV Overlapping	5′-ACGTAAACCCATtctagagagata Gatttgtagagagagactggtgat	5′-ccagtgccaagctgggcatggt ggagcacgacactctc	60°C	784bp
GUS-Overlapping	5′-gactcgacagtctagaATGGGTTTAC G TCCTGTAGAAACC	5′-tccttatagtccatggtaccTCA TTG TTTGCCTCCCTGCTGC	58°C	1812bp
